# Genome Sequence of *Porphyromonas gingivalis* Strain A7436

**DOI:** 10.1128/genomeA.00927-15

**Published:** 2015-09-24

**Authors:** Ryan P. Chastain-Gross, Gary Xie, Myriam Bélanger, Dibyendu Kumar, Joan A. Whitlock, Li Liu, William G. Farmerie, Hajnalka E. Daligault, Cliff S. Han, Thomas S. Brettin, Ann Progulske-Fox

**Affiliations:** a Department of Oral Biology, University of Florida, Gainesville, Florida, USA; b Center for Molecular Microbiology, University of Florida, Gainesville, Florida, USA; c Interdisciplinary Center for Biotechnology Research, University of Florida, Gainesville, Florida, USA; d Bioenergy and Biome Sciences, Bioscience Division, Los Alamos National Laboratory, Los Alamos, New Mexico, USA

## Abstract

*Porphyromonas gingivalis* is strongly associated with periodontitis. *P. gingivalis* strain trafficking and tissue homing differ widely, even among presumptive closely related strains, such as W83 and A7436. Here, we present the genome sequence of A7436 with a single contig of 2,367,029 bp and a G+C content of 48.33%.

## GENOME ANNOUNCEMENT

*Porphyromonas gingivalis* is an oral bacterium that is associated with periodontal disease (1) and multiple systemic diseases (2–5). Importantly, *P. gingivalis* strains demonstrate a variety of pathogenic phenotypes *in vitro* and *in vivo* (6–9), whose genetic mechanisms are not entirely known. Presently, genomic sequences of *P. gingivalis* laboratory strains W83, ATCC 33277, TDC60, HG66, and SJD2 are available (10–14). A7436 is an encapsulated strain of *P. gingivalis*, isolated by V. R. Dowell at the Centers for Disease Control and Prevention in Atlanta, Georgia (15). Importantly, strains A7436 and W83 share similar capsule and fimbriae characteristics (16, 17), and both persist intracellularly during infection of human coronary artery endothelial cells (HCAECs) *in vitro* (6). However, A7436 traffics through dissimilar HCAEC intracellular pathways and fails to induce autophagy (6), a mechanism exploited by W83. Additionally, infection of pregnant rats with A7436 causes more severe uterine and placental lesions than infection with either strain W83 or ATCC 33277, due to increased colonization of these tissues by A7436 (18). This study was undertaken to determine the complete genome sequence of A7436 and enable greater understanding of disparate host intracellular trafficking and tissue invasion phenotypes among *P. gingivalis* strains.

*P. gingivalis* strain A7436 was obtained from S. Offenbacher (University of North Carolina, Chapel Hill) and grown as previously described (19). Genomic DNA was obtained using the Wizard gDNA Purification Kit (Promega) and processed to generate shotgun and 3-kb paired-end libraries, which were sequenced using the 454 Life Sciences GS-20 instrument (20) (Roche). 573,205 reads of 148,818,518 bases, with an average read length of 259 bp, were generated.

GS-20 reads were assembled using Velvet version 0.7.63 (https://www.ebi.ac.uk/~zerbino/velvet/) (21) and Newbler version 2.3 (Roche) (20). Gaps between contigs were closed by editing in Consed (http://www.phrap.org/consed/consed.html) (22–24) and by PCR-augmented Sanger sequencing. The genome was annotated using the RAST (http://metagenomics.anl.gov) (25) and IMG-ER servers (http://img.jgi.doe.gov/er) (26), then amended using Gene Prediction Improvement Pipeline software (https://geneprimp.jgi-psf.org) (27).

The genome of *P. gingivalis* A7436 has approximately 57-fold coverage and contains a single contig of 2,367,029 bp (G+C content of 48.33%). A total of 2,078 genes were annotated, which included 2,011 predicted coding sequences (CDSs), 53 tRNAs, 12 rRNAs, and 1 transfer-messenger (tmRNA). There are 234 subsystems in the genome. 169 protein metabolism, 164 cofactors, vitamins, prosthetic groups, and pigments, 74 RNA metabolism, 87 DNA metabolism, 96 carbohydrates, and 19 membrane transport subsystem features were observed.

The annotated *P. gingivalis* A7436 genome was compared to *P. gingivalis* strains W83, ATCC 33277, and TDC60 using RAST (25) and IMG-ER (26). All-to-all BLASTp comparisons of predicted protein sequences showed that A7436 possesses 90 strain-specific CDSs, of which 82 are annotated as hypothetical proteins. Genome clustering analysis of functional profiles suggests that A7436 is closely related to W83, as indicated by similar fimbriae and capsule characteristics (16, 17). However, the A7436 genome contains a 1.5 Mbp chromosomal inversion that may contribute to its distinct phenotypes (6).

The availability of the A7436 genome expands our ability to compare observed behavior with genotype in a growing number of *P. gingivalis* strains.

### Nucleotide sequence accession number.

This genome sequencing project was deposited in GenBank under accession no. CP011995. The version described is the first version.
